# Interacting Effects of Phenotypic Plasticity and Evolution on Population Persistence in a Changing Climate

**DOI:** 10.1111/j.1523-1739.2010.01552.x

**Published:** 2011-02

**Authors:** THOMAS E REED, DANIEL E SCHINDLER, ROBIN S WAPLES

**Affiliations:** * School of Aquatic and Fishery Sciences, University of Washington1122 NE Boat Street, Seattle, WA 98105, U.S.A.; † National Marine Fisheries Service, Northwest Fisheries Science Center2725 Montlake Boulevard E., Seattle, WA 98112, U.S.A.

**Keywords:** adaptation, climate projections, ecological and evolutionary dynamics, extinction, phenotypic plasticity, adaptación, dinámica ecológica y evolutiva, extinción, plasticidad fenotípica, proyecciones climáticas

## Abstract

**Abstract:**

Climate change affects individual organisms by altering development, physiology, behavior, and fitness, and populations by altering genetic and phenotypic composition, vital rates, and dynamics. We sought to clarify how selection, phenotypic plasticity, and demography are linked in the context of climate change. On the basis of theory and results of recent empirical studies of plants and animals, we believe the ecological and evolutionary issues relevant to population persistence as climate changes are the rate, type, magnitude, and spatial pattern of climate-induced abiotic and biotic change; generation time and life history of the organism; extent and type of phenotypic plasticity; amount and distribution of adaptive genetic variation across space and time; dispersal potential; and size and connectivity of subpopulations. An understanding of limits to plasticity and evolutionary potential across traits, populations, and species and feedbacks between adaptive and demographic responses is lacking. Integrated knowledge of coupled ecological and evolutionary mechanisms will increase understanding of the resilience and probabilities of persistence of populations and species.

## Efectos Interactivos de la Plasticidad Fenotípica y Evolución sobre la Persistencia Poblacional en un Clima Cambiante

El cambio climático afecta a organismos individuales alterando su desarrollo, fisiología, comportamiento y adaptabilidad y a las poblaciones alterando la composición genética y fenotípica, las tasas vitales y su dinámica. Buscamos clarificar cómo están ligadas la selección, la plasticidad fenotípica y la demografía en el contexto del cambio climático. Con base en la teoría y los resultados de estudios empíricos recientes de plantas y animales, creemos que la tasa, tipo, magnitud y patrón espacial del cambio abiótico y biótico inducido por el clima; el tiempo generacional y la historia de vida del organismo; la extensión y tipo de plasticidad fenotípica; la cantidad y distribución de la variación genética adaptativa en espacio y tiempo; el potencial de dispersión; y el tamaño y la conectividad de subpoblaciones son los aspectos ecológicos y evolutivos relevantes para la persistencia de las poblaciones a medida que cambia el clima. Se carece de entendimiento de los límites de la plasticidad y potencial evolutivo de caracteres, y las retroalimentaciones entre respuestas adaptativas y demográficas. El conocimiento integral de mecanismos ecológicos y evolutivos acoplados incrementará el entendimiento de la resiliencia y de las probabilidades de persistencia de las poblaciones y especies.

## Introduction

The persistence of populations and species in the face of environmental change is ultimately shaped by dynamic and often complex feedbacks between ecology and evolution (Kinnison & Hairston 2007). Ecological factors include direct and indirect effects on births, deaths, dispersal rates, and species interactions, whereas evolutionary factors include changes in the genetic and phenotypic constitution of populations (Parmesan 2006). Typically, ecological and evolutionary responses to environmental change are considered separately (Ferrière et al. 2004; Kokko & Lopez-Sepulcre 2007; Pelletier et al. 2009). Evolution, however, often occurs rapidly and can influence contemporaneous ecological dynamics (Hairston et al. 2005). Global climate change will not only affect migration patterns, biotic interactions, and local population dynamics, but also the selective pressures experienced by populations. Coupled ecological and evolutionary changes are therefore expected under climate change.

Ignoring evolutionary processes can produce an incomplete picture of likely biotic responses to climate change (Holt 1990; Davis et al. 2005; Visser 2008). For example, most projections of species responses to climate change are based on niche models, which assume that a species has a single, static environmental tolerance function. Regional populations, however, are often locally adapted to a more limited range of conditions (e.g., temperatures) than that experienced by the species as a whole. Overlooking regional differentiation can lead to overestimates of probabilities of population persistence (Davis et al. 2005; Etterson 2008) or erroneous projections of poleward or elevational range expansions if local adaptation in peripheral populations is disrupted (Pelini et al. 2009). Conversely, by overlooking the possibility that environmental tolerances might evolve or increase through phenotypic plasticity, static niche models can overestimate probabilities of extinction or range contraction (Holt & Gomulkiewicz 2004; Davis et al. 2005).

Realistic assessment of probabilities of population and species persistence thus requires information on both current levels of adaptation and future adaptive potential (Davis et al. 2005; Etterson 2008). Additionally, evolutionary assessments must incorporate key ecological considerations, such as the demographic consequences of strong selection in closed populations (Lynch & Lande 1993; Gomulkiewicz & Holt 1995) or metapopulations (Holt & Gomulkiewicz 2004), the modulating effects of phenotypic plasticity on population and evolutionary dynamics (Price et al. 2003), and potential feedbacks between selection and population regulation (Kinnison & Hairston 2007; Kokko & Lopez-Sepulcre 2007).

Determining limits to phenotypic plasticity and evolutionary potential in traits affecting survival, reproduction, and dispersal will be critical to assessing demographic responses to climate change (Hellmann & Pineda-Krch 2007; Bradshaw & Holzapfel 2008; Gienapp et al. 2008; Visser 2008). We attempted to clarify how evolution, phenotypic plasticity, and demography are linked in the context of climate change.

## The Adaptive Surface

Fitness landscapes are heuristic tools that represent the fitness consequences of major environmental changes (e.g., Hellmann & Pineda-Krch 2007). Individual-level fitness (adaptive) surfaces illustrate the relation between phenotype (measurable traits of an organism) and relative fitness. The topography of this surface reflects the probable lifetime reproductive success of individuals with different phenotypic characteristics in that environment. Further an individual's phenotype is from the optimum, the lower its relative fitness. If population mean fitness (average number of offspring produced per capita per time period, also called absolute fitness) is plotted against a range of hypothetical mean trait values, the resulting surface is called a phenotypic adaptive landscape, which can have one or several “adaptive peaks” (fitness optima).

If the mean phenotype does not correspond to an adaptive peak (i.e., if local adaptation is imperfect), mean absolute fitness is reduced relative to the optimum, and the population experiences directional selection uphill toward the nearest peak (Lande 1976). Hereafter, we consider simple adaptive landscapes that are characterized by a single adaptive peak, even though real environments are generally more complex (Arnold et al. 2001). Deviation of the mean phenotype from the optimum is called the lag, whereas the relative decrease in mean fitness resulting from the lag is called the lag load, or evolutionary load (Lynch & Lande 1993; Bürger & Krall 2004).

Climate change can alter fitness landscapes in several ways. Suboptimal temperatures or changes in other key variables could increase mortality or lower fecundity independent of phenotype, which would effectively lower the overall elevation of the fitness surface (improved conditions would do the opposite). At the same time, climate change can shift the location of the phenotypic optimum through trait space (e.g., Grant & Grant 2002) and potentially alter the shape of the fitness surface (e.g., Charmantier et al. 2008). If the optimum changes consistently through time but the mean phenotype lags behind, extreme phenotypes closer to the optimum will have consistently higher fitness and the population will experience increasingly strong directional selection. Alternatively, if fluctuations in the optimum about a fixed or slowly changing mean become stronger or more frequent, the population will experience selection that varies increasingly in intensity and possibly also in direction (Bürger & Krall 2004; Hellmann & Pineda-Krch 2007).

Even if environmental change does not affect the phenotypic optimum, selection pressures could change if maladaptive plasticity causes individuals to produce phenotypes that are locally suboptimal. If the mean phenotype deviates consistently from the optimum, the resulting maladaptation might depress population growth below replacement level. Unless demographic rescue from neighboring populations occurs, there are only three options: move, adapt, or die (Pease et al. 1989; Lynch & Lande 1993).

Extant species have persisted through numerous past climate shifts. Two phenomena can facilitate adaptive tracking of temporal changes in the environment: adaptive evolution (changes in population gene frequencies across generations that result in phenotypes that are more likely to persist in new environments) and adaptive phenotypic plasticity (fitness-enhancing responses by individuals to environmental cues). Plastic phenotypic responses do not involve genetic change at the population level, but the capacity for plasticity itself can be adaptive and genetically variable within populations, and this capacity may evolve in response to changes in environmental variability (Scheiner 1993; Svanbäck et al. 2009). Whether populations can persist depends on the nature, magnitude, and rate of change in selective pressures, the capacity for evolutionary and plastic (including migration) responses, and the demographic context of adaptation.

## Natural Selection and Adaptive Evolution

Climate variation affects ecological processes at multiple scales and influences local selection pressures. For example, in finches endemic to the Galápagos Islands (*Geospiza fortis* and *G. scandens*), changes in rainfall driven by the El Niño-Southern Oscillation affect relative abundance of seeds of different sizes and hardness, which in turn drive fluctuating patterns of selection on body size and beak morphology (Grant & Grant 2002). In a changing climate, selection can shift from stabilizing, where intermediate trait values have highest fitness, to net-directional. Alternatively, local climates might become more variable and extreme weather events more frequent (Easterling et al. 2000), in which case populations experience selection that fluctuates more in sign and magnitude. The latter is expected to favor increased plasticity, although several factors might limit evolutionary changes to phenotypic plasticity.

Predicting changes in natural selection requires identifying biotic and abiotic factors exerting selective pressures, estimating likely temporal and spatial changes in these factors under different scenarios of climate change, identifying which traits, or combinations of traits, will be targets of this selection, and determining how phenotypic plasticity affects (potentially correlated) trait selection.

Extrapolating selection patterns in future environments necessitates knowledge of mechanistic links between climate variation, phenotypes, and fitness (Holt 1990). Studies on Great Tits (*Parus major*) and trophic interactions in the Netherlands, where spring temperatures have been rising gradually, are illustrative. Since 1973 researchers measured annual selection differentials (a measure of the strength of directional selection, defined as the covariance between trait and relative fitness) for the date egg laying began and documented increased selection for earlier egg laying (Visser et al. 1998). The ecological mechanism underpinning the changing relation between trait and fitness involves shifts in the phenology of food (larvae of the winter moth [*Operophtera brumata*]) for chicks and potential effects of changing larval phenology on the productivity of Great Tits (Visser et al. 2006). Timing of egg hatching in *O. brumata* is itself under selection for synchronization with bud burst in oak trees (*Quercus robur*). Caterpillars of this moth rely on oak leaves for food, and although the oak trees have been opening their buds earlier, advancement in the date of egg hatching has been more extreme (van Asch et al. 2007). Caterpillars now emerge before their food peaks and consequently are most available for Great Tits earlier in the season (Visser et al. 2006).

For evolution to occur, at least some of the variation among individuals in traits affecting survival and reproductive success must be transmitted from parents to offspring. Given knowledge of selective pressures and genetic variances and covariances (inheritance patterns underlying trait variation), the expected trajectory and rate of evolutionary change can be calculated (Price 1970; Lande & Arnold 1983; Falconer & Mackay 1996). Selection acts on expressed phenotypes and both the expression of genetic variation and the strength of selection often depend on the environment (Kruuk et al. 2008). Obtaining estimates of selection intensity and quantitative genetic parameters across a range of environmental conditions allows for more accurate prediction of how mean traits values might respond to climate change (Etterson 2008).

Under continual directional selection, the mean phenotype will lag behind a moving optimum because traits are never 100% heritable. The key issue is to determine whether the resulting lag load is demographically sustainable. Extinction probability following a large, abrupt environmental change can be substantial in populations of low to medium density even if they exhibit adaptive evolution after the change (Gomulkiewicz & Holt 1995; but see Boulding & Hay 2001). This is because strong directional selection generates a large number of deaths each generation. When environmental change is gradual, evolving populations can track a moving selective optimum, but persistence still depends on the continued availability of sufficient genetic variation in the right phenotypic direction (i.e., in a direction aligned with that of selection) (Lande & Arnold 1983; Bürger & Krall 2004; Jones et al. 2004).

Whenever selection acts on multiple phenotypic traits simultaneously, genetic correlations among traits, or a lack of genetic variance for some, can prevent or slow the rate of evolution toward the optimum combination of trait values (i.e., the one which would yield the highest fitness) (e.g., Dickerson 1955; Lande & Arnold 1983; Via & Lande 1985). For example, if two traits are negatively correlated genetically but selection favors higher phenotypic values for each, then an increase in one would result in a decrease in the other, and consequently neither might evolve (Etterson & Shaw 2001). The greater the opposition between trait genetic covariance and the direction of multivariate selection, the faster the population accrues a lag load in a monotonically changing environment (Hellmann & Pineda-Krch 2007). The resulting drop in absolute fitness can lead to reductions in abundance and make a population more susceptible to genetic and demographic stochasticity (Bürger & Krall 2004).

Conversely, genetic correlations between traits that match the direction of selection can increase the rate of evolution (relative to the situation in which traits are independent (Agrawal & Stinchcombe 2009) and potentially reduce relative extinction probability. Absolute extinction probability might still increase if environments become increasingly stochastic or if carrying capacity is reduced. Most biologists agree that selection is unlikely to act on traits in isolation, yet few have quantified genetic covariation among traits affecting individual fitness in the context of an altered climate (Hellmann & Pineda-Krch 2007).

Several classes of traits are expected to experience strong selection in general as climate changes, such as traits related to the timing of life-history events, physiological tolerances, dietary preferences, and disease resistance. Bradshaw and Holzapfel (2008) argue that timing of key life-history transitions, such as timing of reproduction, will often be under strongest selection. Evolutionary responses to longer growing seasons have already been documented in a range of animals, including mosquitoes, squirrels, and migratory birds (Bradshaw & Holzapfel 2008). Longer summers and shorter winters might also lead to prolonged droughts in some regions, which would select for phenological changes in plants. For example, between 2000 and 2004, low winter precipitation led to shorter growing seasons for *Brassica rapa* (field mustard) in southern California. Thus, earlier flowering was selected for and many late bloomers senesced before they produced seeds. Plants bloomed 8.6 days earlier in one population following the drought, and survival of postdrought genotypes was higher than predrought genotypes (grown from stored seed) when both were grown under conditions mimicking a shorter season (Franks et al. 2007).

Rising temperatures, atmospheric CO_2,_ and ocean acidity will impose strong and complex selection on terrestrial and aquatic organisms to adapt their physiology (Pörtner & Farrell 2008). The degree to which different aspects of thermal physiology are evolutionarily labile within and across taxa is debated (Angilletta et al. 2002), but genetic constraints (e.g., negative genetic correlations between performance at high and low temperatures or between different aspects of performance) may be a real limitation for some populations. Heat tolerance in ectothermic animals may be under weaker selection than cold tolerance (Bradshaw & Holzapfel 2008). Nonetheless, intensified selection on thermal performance may occur in many situations in the future. In the tropics, terrestrial ectotherms already live close to their physiologically determined thermal optima and have much narrower thermal tolerances compared with ectotherms that live at higher latitudes (Deutsch et al. 2008). Although greater warming is predicted at higher latitudes (IPCC 2007), relatively small temperature increases or increased thermal variability in the tropics could affect fitness in ectotherms greatly and select for higher or broader temperature tolerances. Additionally, the fitness of ectotherms in warming temperate regions may initially increase (Deutsch et al. 2008). Increased thermal variability may be detrimental for aquatic species, however, whose capacity for aerobic performance decreases substantially at both low and high temperatures, where oxygen supply becomes limiting (Pörtner & Farrell 2008).

In the oceans rising temperatures will increasingly impose thermal stress on corals, and models suggest persistence will require an increase in the thermal tolerance of corals of 0.5–1 °C by 2060 (Donner et al. 2005). Acclimatization responses such as the uptake of novel heat-tolerant symbionts (*Symbiodinium* spp.) may partially increase tolerance, but genetic responses in the host or symbiont would likely also be necessary (Baird et al. 2009).

Ocean acidification resulting from increased dissolution of atmospheric carbon dioxide (forming carbonic acid) inhibits the ability of hard-shelled marine organisms, including reef-building corals, to incorporate calcium carbonate into their shells. Although mechanisms allowing more efficient extraction of carbonates from seawater may evolve, pH could decrease so much that exoskeletons dissolve. For all organisms, rapid adaptation of physiological tolerances to climate change remains plausible but uncertain.

## Causes, Consequences, and Limits of Phenotypic Plasticity

Plastic phenotypic responses by individuals to shifting conditions, such as changes in behavior (including migratory patterns), physiology, and morphology, provide another means to persist as climate changes. Phenotypic plasticity, the environment-dependent expression of phenotypes by genotypes, can substantially alter the phenotypic distribution (mean and variance) of populations over time without a genetic change. Plasticity is evolutionarily favored when the environment is heterogeneous in time or space, selection favors different phenotypes in different environments, no one phenotype has greatest fitness across all environments, and reliable cues allow organisms to respond effectively (Bradshaw 1965; Via & Lande 1985; Gomulkiewicz & Kirkpatrick 1992; Scheiner 1993). Costs associated with acquiring environmental information, producing different phenotypes, and maintaining the physiological and developmental capacity for facultative responses, however, can substantially constraint the evolution of phenotypic plasticity (Scheiner 1993; DeWitt et al. 1998; de Jong 1999).

Adaptive plasticity in key traits may allow populations to track shifting selection pressures without much evolution. For example, warmer, drier springs in the Canadian Yukon have led to an increase and seasonal advancement in cone production of white spruce (*Picea glauca*) the primary food source for North American red squirrels (*Tamiascurus hudsonicus*). In response, female squirrels have advanced their parturition dates by approximately 18 days over a 10-year period, which has occurred mostly through plasticity rather than genetic adaptation (Réale et al. 2003). Mean lifetime reproductive success has remained stable, which suggests the squirrels have remained well adapted (Berteaux et al. 2004; Charmantier et al. 2008).

Plasticity has limits, however (DeWitt et al. 1998), and once these are exceeded adaptive evolution or demographic rescue from neighboring source populations (populations in which the number of births exceeds the number of deaths) provide the only mechanisms for persistence. The limits to adaptive plasticity in wild populations are not well understood. Empirical studies relating phenotypic responses of known individuals to environmental changes can increase understanding, particularly if fitness consequences can be deduced (e.g., Réale et al. 2003; Nussey et al. 2005; Charmantier et al. 2008). Understanding also can be increased by experiments in which temperatures or other environmental variables are manipulated beyond the historical range. Plastic and evolutionary responses to the resulting artificial selection can be determined statistically with reciprocal-transplant experiments (e.g., Potvin & Tousignant 1996) or experiments with pedigreed families (Etterson 2008).

Reaction norms are functional relations that describe how traits change as a result of phenotypic plasticity when the environment changes. Regardless whether plasticity limits are exceeded, selective pressures on reaction norms will almost certainly change as climate and ecological interactions change. Simple, linear reaction norms are characterized by two parameters: elevation, which describes the expected phenotype in the average environment, and slope (degree of plasticity), which measures how responsive the phenotype is to environmental change. Independent selection pressures on each component are difficult to predict, but several broad scenarios can be distinguished on the basis of expected environmental change.

In many ecosystems, particularly at high latitudes, varying rates of phenological and ontogenetic changes across species are leading to mismatches between interacting species (e.g., predators and prey). In situations where environmental cues used by organisms to time life-history transitions no longer are effective, selection might favor changes in reaction—norm elevation, shape (e.g., slope), or both. For example, caterpillar prey for Great Tits in the Netherlands are now available to feed chicks considerably earlier in the summer than several decades ago (Visser et al. 1998). Females who always lay early and those who lay early only in warm years (more plastic females), have higher reproductive success (Nussey et al. 2005). Variation across females in these reaction-norm components (elevation and slope) has a heritable component, which suggests that reaction-norm evolution could alleviate the negative effects of phenological shifts for this population (Nussey et al. 2005).

If the frequency of previously rare extreme climatic events increases (Easterling et al. 2000), selection pressures and population dynamics may become more erratic, and this may favor evolution of increased plasticity (Svanbäck et al. 2009). Nevertheless, if plasticity entails substantial energetic costs that reduce fitness, selection is expected to favor generalist (high plasticity) strategies only when ecological dynamics are highly variable or cyclic. Otherwise, selection should favor low-cost specialist (low plasticity) strategies (Svanbäck et al. 2009). Even if energetic costs of plasticity are low, stronger plasticity will be selected for only if environments remain predictable, which is unlikely (Visser 2008). Unless organisms can switch to more reliable cues, selection may favor reduced plasticity (Scheiner 1993; de Jong 1999)— even if environments become more variable.

Moreover, local environments may change rapidly, for example, due to an abrupt climatic shift or the appearance of a novel predator. Sudden, qualitative shifts in abiotic conditions or community composition can render previously adaptive plastic responses suboptimal (Langerhans & DeWitt 2002). Under these circumstances, selection may favor phenotypes beyond the range that can be produced by nonplastic genotypes. If genetic variation for plasticity exists in the population and some plastic genotypes produce phenotypes that are better adapted to the new conditions, directional selection may favor these induced extremes (Badyaev 2005). Such a process of plasticity and subsequent “genetic assimilation” (eventual flattening of reaction norms) may allow populations to evolve rapidly enough to survive abrupt change (Price et al. 2003; Lande 2009).

Higher environmental stress may also increase mutation or recombination rates, thereby increasing genetic diversity and evolutionary potential (Hoffmann & Parsons 1997). Phenotypic diversity may be further enhanced if “cryptic genetic variation” is released, that is, variation outside the normal range of reactions that was never expressed phenotypically (McGuigan & Sgro 2009). If either or both of these processes lead to the emergence of novel phenotypic variants that perform well under the novel stresses, adaptive evolution may occur fast enough to preclude extinction (Carroll & Watters 2008).

## Merging Evolutionary and Ecological Considerations

Experimental studies that explore fitness consequences of phenotypic and genetic change under simulated climate change in different demographic contexts (e.g., large vs. small populations or closed populations vs. metapopulations) can test theoretical models of extinction risk and elucidate general, adaptive mechanisms and genetic and demographic constraints that might affect population and species persistence in the wild.

Theoretical models suggest maximum sustainable rates of continuous evolution in closed populations could be <10% of a phenotypic standard deviation per generation. Any continuous shift in the optimal phenotype beyond this rate may overwhelm evolutionary potential and decrease abundance (Lynch & Lande 1993; Bürger & Krall 2004). These models assume adaptation occurs gradually by successive substitution of alleles (gene variants) with mostly small phenotypic effects. Nevertheless, adaptation to novel environments often involves mutations that have large effects (Keeling et al. 2008). More recent models suggest that different combinations of genetic architecture (e.g., number and effect size of genes affecting the trait; frequency of occurrence of major mutations) and types of environmental change (e.g., sudden vs. gradual) can have complex consequences for rates of adaptation and population persistence (Kopp & Hermisson 2007; Gomulkiewicz et al. 2010). Adaptive plasticity will also reduce the demographic impact of strong selection, allow more time for evolutionary adaptation, and reduce the amount of evolutionary change necessary to track a moving optimum (Chevin et al. 2010). Simulation models that capture essential evolutionary and ecological dynamics (e.g., Boulding & Hay 2001; Holt & Gomulkiewicz 2004) could be extended to incorporate plasticity and alternative modes of genetic architecture and then used to explore different climate-change scenarios.

Altered patterns of gene flow between open populations (or between species at hybrid zones) may play an important role in modulating adaptive responses to climate change. Increased gene flow could lead to an influx of useful genetic variation that might permit faster evolutionary tracking of environmental change or facilitate shifts between adaptive peaks. Conversely, gene flow from populations adapted to very different conditions could disrupt local adaptation (e.g., immigrants might not have resistance to local strains of parasites). For example, increased regional climate heterogeneity could accentuate differences in selective regimes across a species’ range, which could increase migration loads (the reduction in mean fitness because of immigration of maladapted genotypes that breed and compete with local genotypes). Under such circumstances, adaptation and persistence may be maximized at intermediate dispersal levels, although the advantages of immigration may outweigh its negative effects when populations are initially adapting to locations that have become demographic sinks (Holt & Gomulkiewicz 2004).

For organisms with limited dispersal capabilities, habitat fragmentation and climate change may together render long-term persistence unlikely (Travis 2003), unless in situ adaptation can keep pace with environmental changes. Species with shorter generation times, higher initial population sizes, and higher fecundity are expected to evolve faster for a given absolute pace of physical change. Increased climatic variability may exceed the current capacity of long-lived species with low fecundity to persist through phenotypic plasticity (e.g., via behavioral buffering of adult survival) (Forcada et al. 2008). Many threatened species are long-lived and have small population sizes, whereas species currently considered invasive may adapt most readily.

Establishing the rates of climate change that allow population persistence via phenotypic plasticity and evolution requires identifying which aspects of climate shape the selective pressures and currently limit the abundance and distribution of organisms (Holt & Gomulkiewicz 2004; Helmuth et al. 2010). Conservation measures, such as effort to increase the quantity, quality, and connectivity of a species’ habitat, could at least maintain if not be targeted to increase phenotypic and genetic diversity (and therefore adaptive potential) within and among populations (Carroll & Watters 2008).

Most current projections of extinction probability as climate changes (e.g., Thomas et al. 2004; Malcolm et al. 2006) do not account for adaptation. Conservation plans that aim to preserve key ecological and evolutionary processes, rather than status quo phenotypes, will at least ensure that species have a fighting chance against climate change.
